# Massive Chronic Hypernatremia Associated With Failure to Thrive in a Pediatric Patient

**DOI:** 10.7759/cureus.42179

**Published:** 2023-07-20

**Authors:** Jack A Tellerday, Vincent Calleo

**Affiliations:** 1 Emergency Medicine, Upstate University Hospital, Syracuse, USA; 2 Toxicology, Upstate University Hospital, Syracuse, USA

**Keywords:** community-acquired hypernatremia, hypernatremia, inpatient regimens, fluid management, pediatric neurology, pediatrics emergency

## Abstract

Hypernatremia is a severe, potentially life-threatening condition that can manifest with altered mental status, coma, seizure, and even death. Values above 190 mmol/L are seldom reported in young pediatric patients and often have poor outcomes. We present a case of severe chronic hypernatremia secondary to failure to thrive (FTT) in a toddler, which led to significant pathology including bilateral metabolic strokes. A 21-month-old female was found unresponsive and brought to the hospital. The patient’s childhood was complicated by prematurity, poor weight gain, and persistent postprandial emesis. On examination, the patient was tachycardic and obtunded. Her weight was below the first percentile. Initial laboratory results showed a sodium level of 197 mmol/L with marked dehydration. Normal saline boluses were given followed by maintenance fluids with the goal of sodium decrementation by 0.5 mmol/hour; nephrology assisted with fluid and electrolyte correction calculations. Imaging revealed metabolic strokes involving the brainstem and thalami. During hospitalization, hypokalemia and hypophosphatemia complicated the treatment course. Over the next 21 days, electrolytes normalized. She tolerated nasogastric feeding, gradually improved as she gained weight, and was discharged. Chronic hypernatremia must be fixed judiciously as rapid correction can cause significant harm. This unusual case reminds providers that florid electrolyte dyscrasias may be secondary to FTT and can lead to significant neurological sequelae. Careful fluid selection and calculations should be performed in these cases. Chronic hypernatremia should be considered in children with FTT with altered mental status, and the gradual correction of electrolytes should be performed to minimize patient harm.

## Introduction

Hypernatremia is typically defined as a serum sodium concentration greater than 145 mEq/L and can manifest with coma, seizure, and death at values over 160 mEq/L. Hypernatremia in children often manifests months after birth, with severe levels occurring with insufficient transfer of breast milk to infants during breastfeeding inducing a state of dehydration [1]. Other notable causes of hypernatremia in children include diarrheal dehydration, diabetes insipidus, salt poisoning, and renal tubular disorders. Values above 190 mEq/L are seldom reported in the literature and are often fatal [2,3]. Prompt efforts must be made to carefully correct the sodium levels; the consequences of hypernatremia are broad and multi-organ system-based. Here, we present a case of severe hypernatremia (197 mEq/L) and failure to thrive (FTT) in a pediatric patient leading to bilateral metabolic stroke.

## Case presentation

A 21-month-old female presented to the hospital for the evaluation of altered mental status and unresponsiveness. The patient’s mother reported finding the patient limp and unresponsive when she went to wake up the child. On arrival, emergency medical services (EMS) noted the patient to be hypotensive, tachycardic, and unresponsive to noxious stimuli. The patient was born prematurely at 33 weeks and five days, and the pregnancy was complicated by preeclampsia and pancreatitis with a two-week neonatal intensive care unit (NICU) stay. The patient’s mother reported that the patient had poor weight gain, developmental delay, and intermittent episodes of poor feeding and projectile emesis after every feed since leaving the NICU.

On the initial physical examination, the patient appeared toxic, emaciated, and obtunded. The patient’s height and weight were markedly below the first percentile for her age. Her skin was pale, and mucous membranes were dry. Dorsalis pedis and radial pulses were barely palpable, and capillary refill was significantly elevated. Systolic blood pressure was palpated at 70 mmHg. Fingerstick glucose was within normal limits at 114 mg/dL. With good oxygen saturation in the high 90s on room air and the patient protecting her airway well, the decision was made to not intubate the patient. Fluids and empiric ceftriaxone were administered. The patient was resuscitated with a total of 60 mL/kg of isotonic fluids in the emergency department (ED) at which point they noted her to become more responsive to stimuli. She remained tachycardic with diminished capillary refill, so she was given another 20 mL/kg bolus of balanced crystalloid fluids with improvement in her peripheral perfusion. Initial laboratory results are summarized in Table 1. The patient’s mother denied any known ingestions that could have contributed to the patient’s severe hypernatremia. The patient was admitted at this time to the pediatric intensive care unit (PICU).

**Table 1:** Initial chemistry profile. The bolded values are outside the reference range. BUN: blood urea nitrogen

Laboratory value	Result (reference range)
Bicarbonate	26 mmol/L (22-29 mmol/L)
Sodium	197 mmol/L (135-145 mmol/L)
Potassium	3.1 mmol/L (3.5-5.2 mmol/L)
Chloride	>140 mmol/L (96-106 mmol/L)
BUN	52 mg/dL (7-20 mg/dL)
Creatinine	0.68 mg/dL (0.5-1.0 mg/dL)
Glucose	245 mg/dL (70-100 mg/dL, fasting)
Calcium	8.0 mg/dL (8.6-10.3 mg/dL)

**Figure 1:**
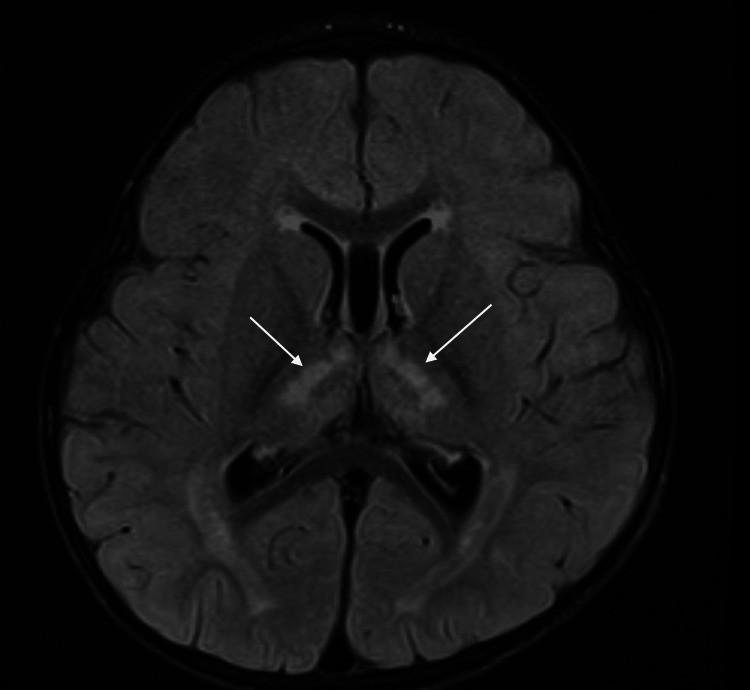
Acute infarction in the left and right thalami with extension inferiorly into the midbrain and pons, T2-FLAIR MRI series. FLAIR: fluid-attenuated inversion recovery

On admission to the PICU, the patient was initiated on hypotonic fluids with 5% dextrose (D5) with 77 mEq of sodium acetate calculated for a slow correction of hypernatremia of approximately 0.5 mEq/L/hour. Neurological examination showed hyporeflexia and depressed neurological state, so an MRI of the brain was performed, which showed an acute infarction in the bilateral thalami, midbrain, and pons (Figure 1).

**Figure 2:**
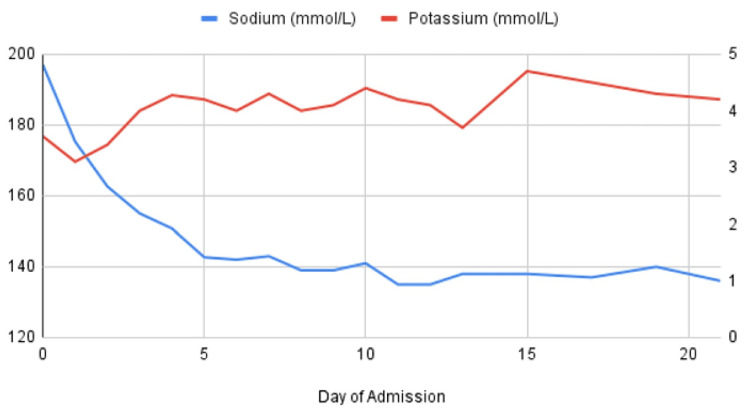
Trend in sodium and potassium values during admission.

Once the patient’s sodium level decreased to 180 mg/dL approximately 24 hours since the patient’s presentation, her fluid regimen was shifted to D5 lactated Ringer’s solution. In the next few days, fluids were adjusted with varying regimens of D5 sodium acetate or normal saline with potassium chloride (KCl), responding to the patient’s persistent hyperchloremia and overall acidosis. During her admission, the patient’s potassium and phosphorus continued to trend down, prompting concern for refeeding syndrome, which was managed with potassium phosphate and various fluid and feeding regimens. Over the course of the patient’s three-week admission, she exhibited many improvements. Her electrolytes normalized (Figure 2); she improved significantly in tolerating feeding from tube feeds to eventually oral (PO) intake. The patient’s neurological examination did not significantly change over the course of the admission, though she did become more responsive and vocal. Reflexes remained diminished at 1+ throughout, and strength did not increase past 3/5 in all extremities. The patient was discharged to follow with neurology on an outpatient basis for repeat MRI and the monitoring of improvement.

## Discussion

Hypernatremia is a common electrolyte disturbance arising from excessive free water loss or excessive sodium intake. Depending on the severity, hypernatremia can present with mortality rates approaching 60% [4]. Various recommendations exist regarding the optimal algorithm to correct hypernatremia. It is generally recommended to have a slow rate of correction (0.5 mmol/L/hour or less) if the patient has had elevated sodium for either long periods of time or an unknown duration, and a quicker pace of correction (1 mmol/L/hour) may be advisable for acute hypernatremia [5]. Our patient had presumed chronic hypernatremia as she had feeding difficulties since birth. This poor oral intake and persistent emesis led to a state of chronic dehydration and volume loss with subsequent hyper-concentration of sodium. Similar pediatric cases with hypernatremic dehydration typically present within months after birth and are often the result of the child being unable to breastfeed adequately or due to the composition of the mother’s breast milk [6,7]. This case is unique in that this patient with failure to thrive presented long after birth with life-threatening hypernatremia once endogenous compensatory mechanisms were no longer adequate, an unusual finding.

The rapid correction of hypernatremia is frequently reported as a cause of increased morbidity and mortality, but this is not always the case. One case report notes a correction greater than 1 mEq/L/hour in a patient with a sodium level of >200 mEq/L to be efficacious, and this led to the survival of the patient [8]. Other larger studies also note that the rapid correction of hypernatremia was not associated with an increased risk of mortality or cerebral edema, in contrast to current guidelines [9-11]. Despite this, there are many signs that the rapid correction of hypernatremia remains problematic. Cerebral edema is a possible complication due to the resultant osmotic balancing shifting water from the intravascular compartment to the parenchyma of the brain [12]. This rapid osmolality alteration dominated by the rapidly decreasing electrolyte concentration within the serum can lead to central nervous system (CNS) symptoms such as seizures and encephalopathy.

The slow correction of hypernatremia (0.5 mEq/L/hour) is the generally accepted guideline [5]. In a study of 18 infants with “severe” hypernatremia who were corrected at a rate of 0.5 mEq/L/hour or less, all tolerated this well with no neurological sequelae (e.g., seizure) [13]. As in this case, the slower rate of correction gives the body’s tissues adequate time to adjust to the changing osmolality and will less likely induce massive shifts of fluid between compartments, and this would not overwhelm cellular transport mechanisms for water and solutes.

Our patient was initially given 60 mL/kg of isotonic fluids while in the ED and subsequently 20 mL/kg balanced crystalloid solution additionally due to her persistent low mentation and hypoperfusion. She was maintained on isotonic fluids during admission, as well as before being switched to hypotonic fluids in the PICU. With the switch to hypotonic fluids, this decrease in the tonicity of the extracellular fluid relative to the tonicity of the parenchymal cells will cause cellular swelling, which can be reversed by the physiological mechanism of regulatory volume decrease (RVD). Hypotonic fluid administration has the potential to outpace the cellular RVD, which can result in cell lysis and tissue damage [14]. Based on the magnitude of this patient’s hypernatremia, it is very possible that this fluid regimen involving continuous hypotonic fluids after an initial resuscitation in the ED had an effect on our patient’s central nervous system, manifesting as an infarction in the bilateral thalami, midbrain, and pons. This same fluid regimen may have not led to symptoms in a patient with a milder hypernatremia. Isotonic saline is recommended against for correction unless there is significant circulatory compromise, but it has been reported to have value in the patients with volume depletion, which is seen in our patient with chronic dehydration due to her inability to feed [14]. In this case, the initial goal of the fluid resuscitation would be to increase the total body water before correcting the sodium rapidly. Therefore, the patient’s volume state and magnitude of sodium elevation need to be taken into consideration before administering hypotonic fluids. The increasing use of point of care electrolyte testing could also be a factor in determining what fluid regimens are useful for resuscitation in the ED before full laboratory value report, as well as tailoring care with regard to the degree of hypernatremia.

Given that our patient had significant developmental delay and neurological delay at baseline, it is hard to say precisely when the infarction occurred and its direct cause. Apart from the correction of hypernatremia too rapidly, infarction is a known sequelae of persistent electrolyte abnormalities. This is a nonspecific finding, as both hypernatremia and hyponatremia have been implicated in an increased risk of stroke and cardiovascular disease, as have the abnormalities of potassium and chloride [15,16]. A hyperosmolar state has the potential to damage the endothelium, and vascular rupture can occur due to retraction from the surrounding brain parenchyma. Furthermore, dehydration is a risk factor for vascular occlusion, and our patient was severely and chronically dehydrated [17]. This would be unlikely to be seen bilaterally, which makes a stroke of metabolic origin more likely. It is then also plausible that this patient’s stroke could have been induced by her chronic state of hypernatremia as opposed to the correction of the imbalance. It is also possible that these two factors together induced the infarction.

Despite the goal of correcting the sodium value by 0.5 mEq/L/hour, it did exceed this during the first few days. Her first 24 hours showed a decrease by 0.71 mEq/L/hour, and the second 24 hours saw a decline by 0.75 mEq/L/hour. She did show some neurological improvement in terms of level of alertness, but she was hyporeflexic and hypotonic at baseline according to her mother, so it is challenging to assess how much her inpatient interventions influenced these aspects of her neurological examination. The course of our patient suggests that initial isotonic fluid resuscitation has value in restoring volume status, which should take place prior to the correction of electrolyte abnormalities.

## Conclusions

Chronic hypernatremia should be considered in children with FTT with altered mental status, and the gradual correction of electrolytes should be performed to minimize patient harm. Our patient’s presentation highlights the importance of neurological examination when evaluating failure to thrive and baseline deficits. The potential for metabolic stroke needs to be kept in mind when large electrolyte deviations are being corrected. The value of isotonic volume resuscitation prior to the correction of electrolyte abnormalities may be significant, and great care should be taken with a multidisciplinary team to determine appropriate fluid regimens. It should also be noted that we should educate families going through the birthing process about signs of FTT and stress the importance of medical attention if that were to occur. While not every case of FTT will lead to florid electrolyte abnormalities and chronic hypernatremia, it should not be overlooked.
